# Interspecific comparison of the fecal microbiota structure in three Arctic migratory bird species

**DOI:** 10.1002/ece3.6299

**Published:** 2020-05-18

**Authors:** Hyunjun Cho, Won Young Lee

**Affiliations:** ^1^ Division of Polar Life Sciences Korea Polar Research Institute Incheon Korea

**Keywords:** Arctic birds, diet, fecal bacteria, feeding behavior, gut microbiota

## Abstract

The gut microbiota of birds is known to be characterized for different species, although it may change with feeding items. In this study, we compared the gut microbiota of birds with different feeding behaviors in the same habitat. We collected fecal samples from three Arctic species, snow buntings *Plectrophenax nivalis*, sanderlings *Calidris alba,* and pink‐footed geese *Anser brachyrhynchus* that are phylogenetically quite distant in different families to evaluate effects of diet on gut microbiota. Also, we characterized the prevalence of fecal bacteria using the Illumina MiSeq platform to sequence bacterial 16S rRNA genes. Our NMDS results showed that fecal bacteria of snow buntings and sanderlings were significantly distant from those of pink‐footed geese. Although all three birds were occupied by three bacterial phyla, Proteobacteria, Firmicutes, and Bacteroidetes, dominant taxa still varied among the species. Our bacterial sequences showed that snow buntings and sanderlings were dominated by Firmicutes and Bacteroidetes, while pink‐footed geese were dominated by Proteobacteria. In addition, the bacterial diversity in snow buntings and sanderlings was significantly higher than that in pink‐footed geese. Our results suggest that insectivorous feeding diet of snow buntings and sanderlings could be responsible for the similar bacterial communities between the two species despite the distant phylogenetic relationship. The distinctive bacterial community in pink‐footed geese was discussed to be related with their herbivorous diet.

## Graphical abstract

We showed that fecal bacteria of snow buntings and sanderlings were distant from those of pink‐footed geese. In addition, the bacterial diversity in snow buntings and sanderlings was significantly higher than that in geese. Our results suggest that diet as well as host phylogeny may affect the gut microbiota of Arctic birds.

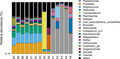

**Concise cover letter**

We collected fecal sample from high Arctic birds, snow buntings and sanderlings (insectivore) and pink‐footed geese (herbivore) and then characterized the prevalence of fecal bacteria. Our results showed that fecal bacteria of snow buntings and sanderlings were distant from those of pink‐footed geese. In addition, the bacterial diversity in snow buntings and sanderlings was significantly higher than that in geese. These results suggest that diet as well as host phylogeny may affect the gut microbiota of Arctic birds.

## 1 INTRODUCTION

Gut microbiota and microbial interactions with the animal can strongly affect host health. Over the past decade, there has been rapid development in investigations into the gut microbiota of vertebrate (Ley, Lozupone, Hamady, Knight, & Gordon, 2008;McFall‐Ngai et al., 2013). In accordance with previous studies demonstrating the significance of vertical transmission in shaping host gut microbial structure, phylogeny of host animals can be a strong predictor of gut microbiota (Kohl, Dearing, & Bordenstein, 2018).

Birds have complex and unique diets, physiological traits, and behavioral strategies. In particular, migratory birds cause unique physiological challenges. For example, migratory birds may have a more complex diet in different habitats. Grond et al (Grond, Ryu, Baker, Domingo, & Buehler, 2014) showed the species‐specific gut microbiota of migratory shorebird species (red knots *Calidris canutus* and ruddy turnstones *Arenaria interpres*) during spring migration staging in Delaware Bay, and more recently, Lewis et al (Lewis, Moore, & Wang, 2017) revealed bacterial changes at stopover sites between spring and fall migrants of Swainson's thrushes (*Catharus ustulatus*) and gray catbirds (*Dumetella carolinensis*) in the Gulf of Mexico. Like other vertebrates, birds harbor diverse microbes in the avian gastrointestinal tract (Roggenbuck et al., 2014;Ryu et al., 2014;Waite & Taylor, 2014). Considering the frequent environmental changes surrounding the migratory birds, it is expected that the gut microbiota would be also affected by the long migration of host animals and the changes could be highly variable among the host species. The broad range of microbial diversity would be related with different habitats along the migratory tracks, and the functional changes of gut microbes would occur under distinctive environmental conditions.

In the Arctic regions, it has high productivity in both plants and insects during the summer season (Callaghan & Jonasson, 1995) and many migratory birds spend its breeding season in this period. North Greenland is a high Arctic region where migratory birds visit to breed in the summer, and waders and geese were found (Boertmann, Olsen, & Nielsen, 2015;Lee, 2018). To examine similarities and differences in bird gut microbiota, we studied three migratory arctic birds: snow buntings (*Plectrophenax nivalis*), sanderlings (*Calidris alba*), and pink‐footed geese (*Anser brachyrhynchus*) during the breeding season in the North Greenland. Those three bird species are phylogenetically distant related each other that belong to order Passeriformes, Charadriiformes, and Anseriformes, respectively. The three orders have different phylogenetic divergence but the divergence time has a long history since the three taxa had been divided: Ancestors of Passeriformes and Charadriiformes diverged from the bird lineage approximately 65 million years ago, and the ancestor of Anseriformes, which belong to Galloanserae, diverged approximately 70 million years ago (Prum et al., 2015). Thus, we assumed that the three orders had enough historical time since divergence so that the autocorrelation among the taxa from the host phylogeny has saturated.

Gut microbiomes generally cluster by host family of vertebrates, including mammals (Groussin et al., 2017;Ley, Lozupone, et al., 2008;Ochman et al., 2010;Phillips et al., 2012), across a wide range of taxa (Coltson & Jackson, 2016). Like other vertebrates, avian gut microbiome is also affected by host phylogeny (Waite & Taylor, 2014). An underlying mechanism for the host‐specific gut microbiota would be through vertical transmission from ancestors shaping similar gut microbiota (Asnicar et al., 2017;Ferretti et al., 2018). Intraspecific variations with geographical region in Adélie penguins (Banks, Cary, & Hogg., 2009) and with the host effect on establishment (Waite & Taylor, 2014) support this idea.

Snow buntings are one of the most northerly passerine birds and predominantly seedeaters, but catch insects for breeding in the summer (Custer & Pitelka, 1975). Sanderlings are a circumpolar breeder in the high arctic, feed on small invertebrates in the intertidal zone by probing, and depend on insects during the breeding season (Castro et al., 2009). Pink‐footed geese feed on green and root parts of plants (Fox, Francis, & Bergersen, 2006). The three bird species migrate to the different regions during winter. Snow buntings may winter in the Russian steppe and sanderlings winter along with Atlantic coasts from the British Isles to Northwestern Africa, and pink‐footed geese move to Iceland and the Britain (Lyngs, 2003). Although we do not have wintering observation records on their dietary behavior, our previous field observations in the summer (in July 2017) indicated that snow buntings and sanderlings were catching insects while pink‐footed geese were foraging plants near the seashore and streams (Lee, 2018).

Host diet is considered as an important factor for describing the gut microbiota that determines the nutritive environment for bacterial growth in the gut of hosts (Colston & Jackson, 2016). The individual shaping of gut microbiomes in the same species can change considerably with its diet (Rothschild et al., 2018). Thus, dietary composition can shape the gut microbial community through many animal species including humans. Also in birds, diet can primarily influence the gut microbiota of birds (Grond, Sandercock, Jumpponen, & Zeglin, 2018).

In this study, high‐throughput sequencing of the 16S rRNA region and a series of statistical analyses were performed to describe microbial community structure and composition and identify the drivers of gut microbiota assemblies. We aimed to elucidate the interspecific comparison in the fecal microbiota in the three Arctic birds. According to the hypothesis that diet would shape the gut microbiota, we predicted that characteristics of the gut bacterial communities, such as diversity, relative abundance of taxa, and community structure, would differ across diet type. If the host taxonomy determined the gut microbiota, we expected that the three birds, which are phylogenetically distant each other, would have different microbial structures.

## 2 MATERIALS AND METHODS

### 2.1 Study site and fecal sample collection

**Figure 1:**
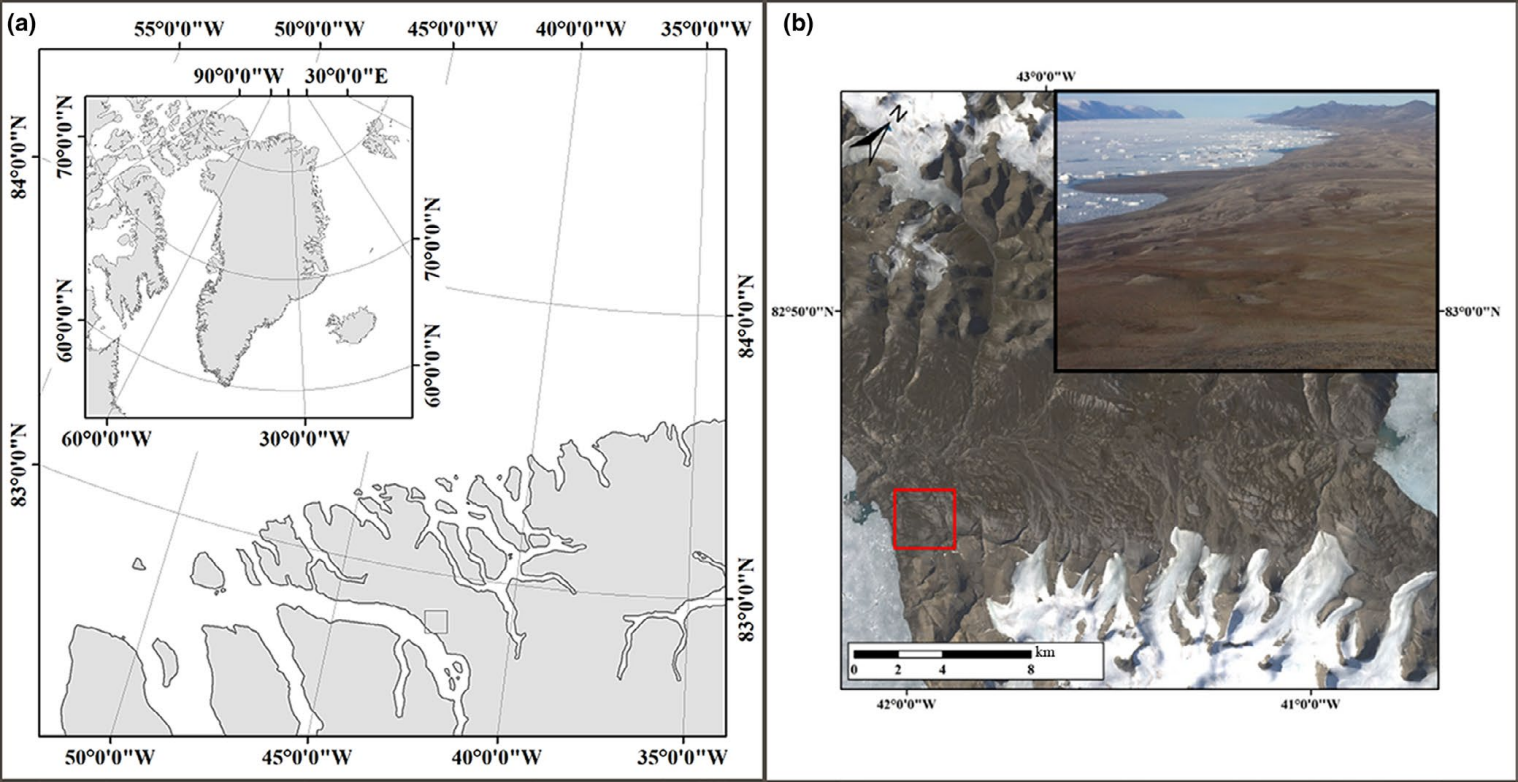
The location of study site. (a) Sirius Passet, at latitude 82° 47' 29.49" and longitude 42° 26' 47.80" W in North Greenland. (b) A detailed satellite image of this study site. Fecal sampling sites are indicated by red square

This study was conducted in a bird colony at J. P. Koch Fjord at the southwestern end of Sirius Passet (82° 47' 29.49" N, 42° 26' 47.80" W) in North Greenland during the 2017 breeding period (June–July) of the three bird species (Figure 1). In 2017, a total of 12 pairs of snow buntings were recorded in rocky areas, and 13 sanderling nests were recorded in flat ground (Lee, 2018). Additionally, pink‐footed geese in small flocks of 20–30 birds were observed near streams and the seashore in mid‐July. We collected 14 samples from three arctic migratory birds from three different orders (Passeriformes, Charadriiformes and Anseriformes), four from the nesting male snow buntings, five from the nesting male sanderlings, and five from the pink‐footed goose which sexes and breeding status were not identified*.* Snow bunting and sanderling were sampled near the bird nests during the breeding and the pink‐footed geese were sampled near the pond. The bird droppings were collected while avoiding the collection of fecal material that was touching the ground to avoid soil contamination using sterile plastic spoons (Yang, Deng, & Cao, 2016).

For fecal sampling, we wore a pair of sterile gloves by spraying 70% EtOH on the gloves and drying them out. Fecal samples, which are widely used in noninvasive proxies for investigating the gut microbiota (Amato et al., 2013;De Filippo et al., 2010;Lewis et al., 2017) were collected from the three arctic bird species. After collection, feces were fixed in a 1.5 ml tube with 99% ethanol solution (Bodawatta, Puzejova, Sam, Poulsen, & Jønsson, 2020;Grond et al., 2019) and filled tubes were placed in a cooler with ice until the end of the day when they were frozen at −20°C until the DNA was extracted.

### 2.2 DNA extraction and PCR amplification

Fecal DNA was extracted using the QIAamp Stool Mini Kit (MoBio Laboratories) following the manufacturer's instructions. The isolated DNA was stored at −80°C until the PCR stage. The V3‐V4 region of the bacterial 16S rRNA gene was amplified using the primers Bakt_341F (5’‐CCTAGGGGNGGCWGCAG‐3’) and Bakt_805R (5’‐GACTACHVGGGTATCTAATCC‐3’) (Huse et al., 2008), and the sequencing primer and adapter sequences for MiSeq. The resultant amplicons were sequenced at Macrogen (Macrogen, INC.) using a paired‐end (2 × 300 nt) Illumina MiSeq sequencing system (Illumina, USA).

### 2.3 Sequencing processing and taxonomic analysis

The sequenced data generated from MiSeq sequencing were processed using the mothur platform (Schloss et al., 2009). The paired‐end sequences of the 16S rRNA gene were assembled using the PANDAseq assembler (Masella, Bartram, Truszkowski, Brown, & Neufeld, 2012). The sequences were aligned against the EzTaxon‐aligned reference (Chun et al., 2007) and further filtered to remove gaps. Sequences were denoised using the “pre.cluster” command in mothur implementation of the pseudosingle linkage preclustering algorithm (Huse, Welch, Morrison, & Sogin, 2010). Putative chimeric sequences were detected and removed via the chimera uchime algorithm contained within mothur in de novo mode (Edgar, Haas, Clemente, Quince, & Knight, 2011). All the 16S rRNA gene sequences were classified against EzTaxon (Kim et al., 2012) using the naïve Bayesian classifier implemented in mothur (at 80% bootstrap cutoff with 1,000 iterations) (Wang, Garrity, Tiedje, & Cole, 2007). The operational taxonomic units (OTUs) were clustered using an average neighbor clustering algorithm with a threshold of ≥97% sequence similarity. All the singleton OTUs were removed from all datasets prior to statistical analysis. After quality filtering, the OTUs allocated as “chloroplast” reads were considered diet contaminants and excluded from all downstream analyses because chloroplast OTUs were not assigned to bacterial phyla. All the 16S rRNA sequence data used in this study are deposited in the MG‐RAST (Meyer et al., 2008) server under project “arctic bird faecal microbiota” (https://www.mg‐rast.org/linkin.cgi?project=mgp90221).

### 2.4 Predicted gut microbiota function using PICRUSt

PICRUSt (Phylogenetic Investigation of Communities by Reconstruction of Unobserved states) v 1.1.0 was used to predict the avian gut microbiome functions and uses an ancestral state reconstruction algorithm to predict metagenomic functional profiles from 16S rRNA gene sequence data and a reference genome database. An OTU table that was produced using a closed reference OTU picking process was used as an input table. The taxonomic information for each OTU was determined using the Greengenes database v13.5 (DeSantis et al., 2006) and then was used to show the relative distribution of shared OTUs. The OTU table was first normalized by 16S rRNA gene copy number predictions and then the metagenomes were predicted and summarized at the level 2 of the KEGG (Kyoto Encyclopedia of Genes and Genomes) classification.

### 2.5 Statistical analysis

All samples were standardized by random subsampling using the “sub.sample” command in mothur to correct for differences in the number of reads between samples. All bacterial sequences were rarified to the lowest number of reads generated from any sample. Rarefaction curve was produced in gplots packge in R version 3.5.1 (R project, http://www.R‐project.org). Sample coverage was calculated in iNEXT package in the R software to estimate the sample completeness by rarefied and extrapolated samples (Hsieh, Ma, & Chao, 2016). Bray–Curtis dissimilarities between all sample pairs were calculated on a square root transformed OTU abundance matrix. The community similarity among all samples was calculated using the Bray–Curtis dissimilarity coefficient and visualized using nonmetric multidimensional scaling (NMDS) conducted in PRIMER6 software (Clarke & Gorley, 2006). Nonmetric multidimentional scaling (NMDS) was used to visualize the differences between bacterial community composition of three arctic bird samples using the “metaMDS” function in the vegan R package (Oksanen, Kindt, Legendre, Minchin, & O’Hara, 2010). Samples were grouped by ellipses enclosing all points in each group using the “ordiellipse” function, and a centroid in the ordination space was calculated to illustrate standard deviations of the community structures in each species in the vegan R package. The ordiellipse function provides ellipsoid hulls of 95% confidence areas by plotting the standard deviations from the centroid (Oksanen et al., 2010). It was used to represent a single ellipse around each cluster in the group by plotting the NMDS results in the ordination. The STAMP program (version 2.1.3) was used to test statistically significant differences between the microbial profiles of three arctic birds (Parks, Tyson, Hugenholtz, & Beiko, 2014), and Welch's *t* test was performed to compare functional profiles from the PICRUSt results (Welch, 1947).

A nonparametric multivariate test (permutational multivariate analysis of variance, which is called “PERMANOVA”) was used to test for differences in bacterial community structure between the three bird species using PRIMER 6 and PERMANOVA+ (Clarke & Tobutt, 2006). Species were included as fixed factors, and p‐values were obtained using 999 permutations. Heat map was generated in ggplot2 package in the R software (Wickham, 2011). We used the invsimpson index to estimate the bacterial diversity and compared the diversity values between the three Arctic birds with one‐way ANOVA and post hoc tests (Tukey's test). The invsimpson diversity values were log‐transformed to satisfy the normal distribution.

## 3 RESULTS

**Figure 2:**
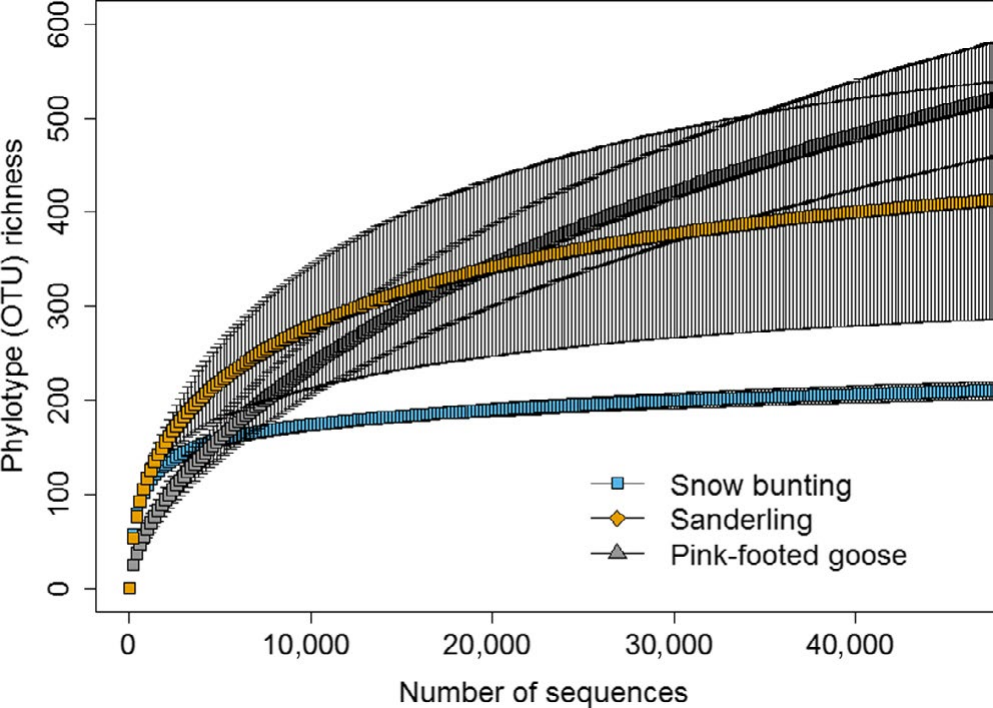
Rarefaction curves (±SE) comparing bacterial communities and the sample coverages were over 99% in the three species (snow buntings: 99.98%; sanderlings: 99.84%; pink‐footed geese: 99.42%)

We obtained a total of 966,547 quality sequences for all fecal samples, with 45,793–72,525 sequences per sample. To correct for differences in the number of reads, all samples were subsampled to the level of the smallest number of reads found in the samples (45,793 reads). The rarefaction curves displayed that it attained the sample coverages were over 99% in the three species by coverage‐based sampling curves at our subsample (45,793 reads) (Figure 2).

**Figure 3:**
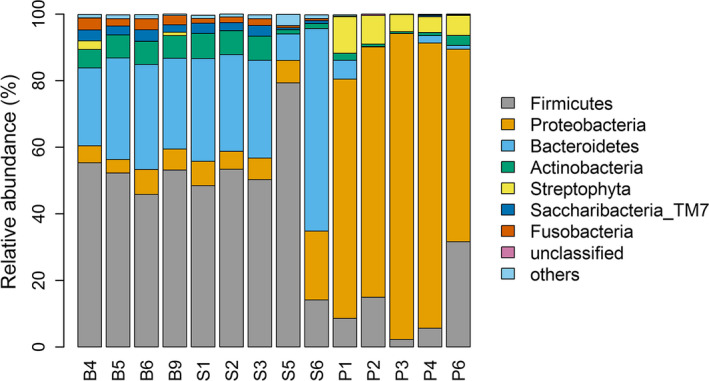
Relative abundances of dominant bacterial phyla of three arctic birds (B4, B5, B6, B9—snow bunting; S1, S2, S3, S5, S6—sanderling; P1, P2, P3, P4, P6—pink‐footed goose)

A total of 3,990 unique OTUs were identified and assigned to more than 50 bacterial phyla. Among the identified groups, the phylum Proteobacteria (38.93%) was the most abundant across all fecal samples (Figure 3) and Firmicutes (31.27%) and Bacteroidetes (18.25%) followed. However, the phylum Proteobacteria was the most abundant in pink‐footed goose, and the phylum Firmicutes was the most abundant in snow bunting and sanderling.

**Figure 4:**
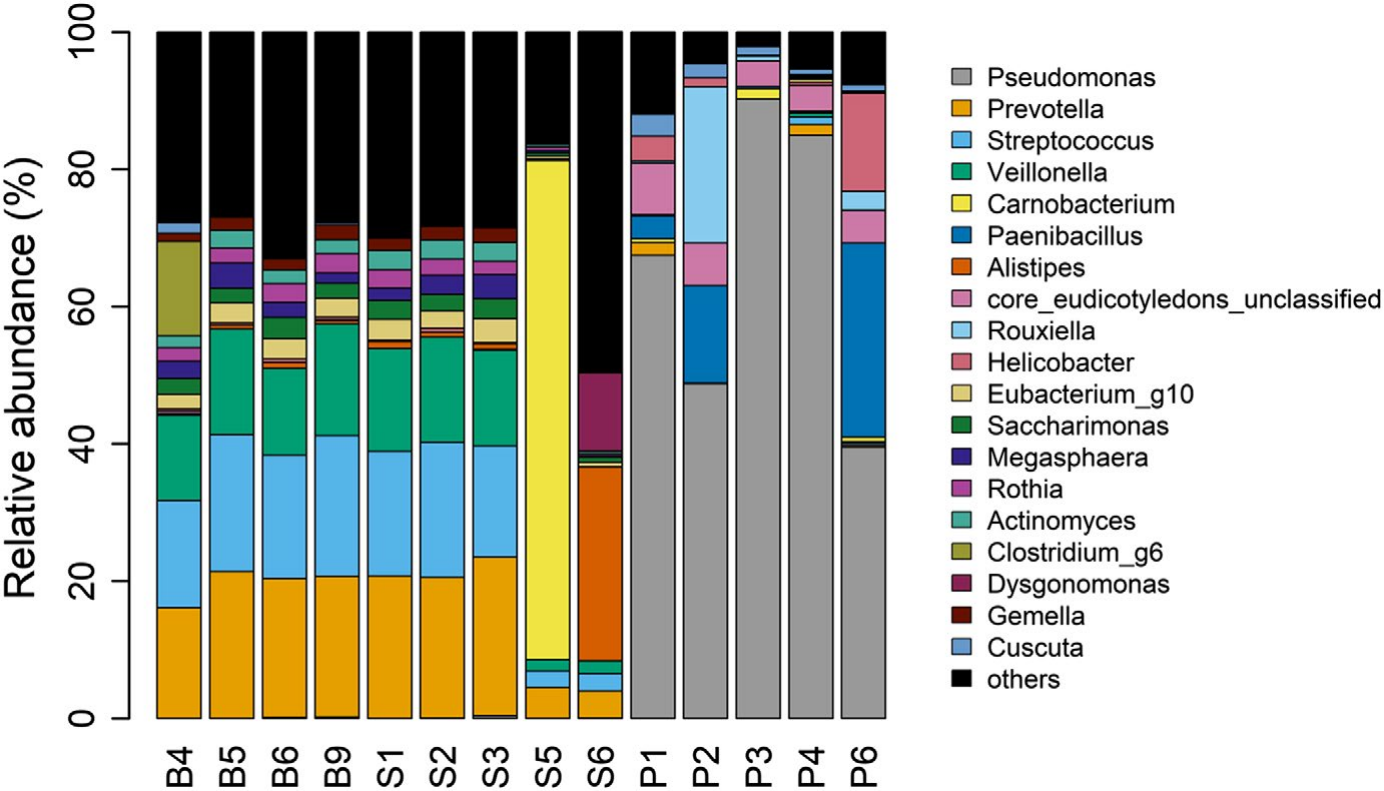
Relative abundances of dominant bacterial genera of three arctic birds (B4, B5, B6, and B9—snow bunting; S1, S2, S3, S5, and S6—sanderling; P1, P2, P3, P4, and P6—pink‐footed goose)

The genus Pseudomonas (66.17%) was the most abundant in pink‐footed goose, and Paenibacillus (9.27%) followed (Figure 4; Table 1). However, the genus Prevotella (17.05%) was the most abundant in snow bunting and sanderling, and the genus streptococcus (15.15%) was the second most abundant (Figure 4; Table 1). The list of most 20 most abundant bacterial genera in snow bunting, sanderling, and pink‐footed goose were provided in Table 1. Among the 20 most abundant genera separately for each species, two genera (Prevotella and Streptococcus) were shared in all three species. Between snow bunting and sanderling, 13 genera were shared (Prevotella, Streptococcus, Veillonella, Eubacterium_g10, Megaspaera, Rothia, Saccharimonas, Actinomyces, Haemophilus, Gemella, Alloprovotella, Fusobacterium, and HM124280_g) while two genera (Prevotella and Streptococcus) were shared between snow bunting and pink‐footed goose and two genera (Prevotella, Streptococcus, and Carnobacterium) were shared between sanderling and pink‐footed goose (Table 1).

**Table 1:** The 20 most abundant bacterial genera (relative abundance, %) in snow bunting, sanderling, and pink‐footed goose

Snow bunting			Sanderling			Pink‐footed goose		
Phylum	Family	Genus	Phylum	Family	Genus	Phylum	Family	Genus
Bacteroidetes	Prevotellaceae	Prevotella (19.54)	Firmicutes	Carnobacteriaceae	Carnobacterium (18.52)	Proteobacteria	Pseudomonadaceae	Pseudomonas (66.17)
Firmicutes	Streptococcaceae	Streptococcus (18.49)	Bacteroidetes	Prevotellaceae	Prevotella (13.34)	Firmicutes	Paenibacillaceae	Paenibacillus (9.27)
Firmicutes	Veillonellaceae	Veillonella (14.22)	Firmicutes	Streptococcaceae	Streptococcus (10.70)	Streptophyta	core_eudicotyledons_unclassified	core_eudicotyledons_unclassified (5.16)
Firmicutes	Clostridiaceae	Clostridium_g6 (3.48)	Firmicutes	Veillonellaceae	Veillonella (8.62)	Proteobacteria	Enterobacteriaceae	Rouxiella (5.07)
Firmicutes	Eubacteriaceae	Eubacterium_g10 (2.69)	Bacteroidetes	Rikenellaceae	Alistipes (6.61)	Proteobacteria	Helicobacteraceae	Helicobacter (3.94)
Bacteroidetes	Prevotellaceae	Prevotella (2.47)	Bacteroidetes	Dysgonamonadaceae	Dysgonomonas (2.62)	Actinobacteria	Sanguibacteraceae	Sanguibacter (0.74)
Actinobacteria	Actinomycetaceae	Rothia (2.45)	Firmicutes	Eubacteriaceae	Eubacterium_g10 (1.82)	Bacteroidetes	Prevotellaceae	Prevotella (0.72)
Saccharibacteria_TM7	Saccharimonas	Saccharimonas (2.43)	Saccharibacteria_TM7	Saccharimonas	Saccharimonas (1.71)	Firmicutes	Carnobacteriaceae	Carnobacterium (0.62)
Actinobacteria	Actinomycetaceae	Actinomyces (2.08)	Actinobacteria	Actinomycetaceae	Actinomyces (1.64)	Firmicutes	Bacillaceae	Bacillus (0.48)
Proteobacteria	Pasteurellaceae	Haemophilus (1.74)	Firmicutes	Veillonellaceae	Megasphaera (15.4)	Firmicutes	Bacillaceae	Psychrobacillus (0.40)
Firmicutes	Gemella	Gemella (1.70)	Actinobacteria	Actinomycetaceae	Rothia (1.44)	Bacteroidetes	Bacteroidaceae	Bacteroides (0.35)
Bacteroidales	Prevotellaceae	Alloprevotella (1.59)	Proteobacteria	Desulfovibrionaceae	Desulfovibrio_g2 (1.43)	Actinobacteria	Microbacteriaceae	Microbacteriaceae_unclassified (0.34)
Fusobacteria	Fusobacteriaceae	Fusobacterium (1.50)	Bacteroidetes	Bacteroidaceae	Bacteroidales_unclassified (1.18)	Proteobacteria	Oxalobacteraceae	Massilia (0.33)
Fusobacteria	Leptotrichiaceae	Leptotrichia (1.47)	Firmicutes	Gemella	Gemella (1.16)	Firmicutes	Clostridiaceae	Clostridium (0.32)
Bacteroidetes	S24−7_f	HM124280_g (1.43)	Bacteroidetes	Rikenellaceae	Rikenellaceae_unclassified (1.14)	Firmicutes	Streptococcaceae	Streptococcus (0.26)
Bacteroidetes	Prevotellaceae	Paraprevotella (1.15)	Bacteroidetes	Prevotellaceae	Alloprevotella (0.93)	Actinobacteria	Micrococcaceae	Arthrobacter (0.20)
Actinobacteriaia	Coriobacteriaceae	Atopobium (1.10)	Proteobacteria	Rhodospirillaceae	LARJ_g (0.89	Proteobacteria	Enterobacteriaceae	Enterobacteriaceae_unclassified (0.20)
Proteobacteria	Moraxellaceae	Moraxellaceae_unclassified (0.98)	Bacteroidetes	S24−7_f	HM124280_g (0.83)	Bacteria_unclassified	Bacteria_unclassified	Bacteria_unclassified (0.17)
Bacteroidetes	Porphyromonadaceae	Porphyromonas (0.96)	Proteobacteria	Pasteurellaceae	Haemophilus (0.79)	Proteobacteria	Gammaproteobacteria_unclassified	Gammaproteobacteria_unclassified (0.16)
Firmicutes	Ruminococcaceae	JN713389_g (0.88)	Fusobacteria	Fusobacteriaceae	Fusobacterium (0.71)	Bacteroidetes	Flavobacteriaceae	Flavobacterium (0.16)

**Figure 5:**
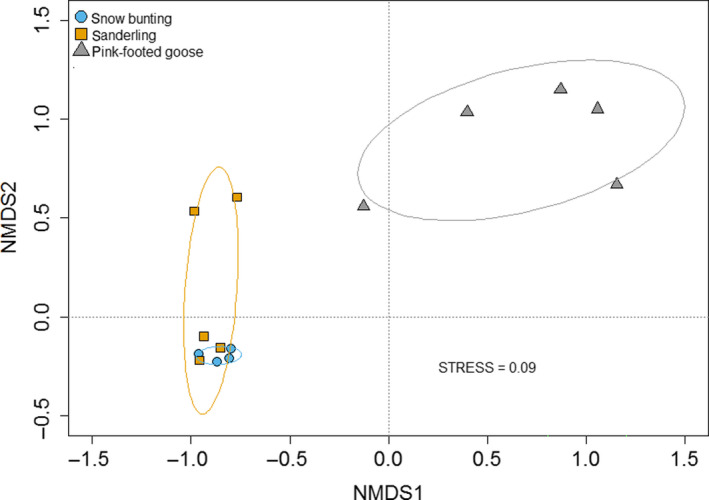
Nonmetric multidimensional scaling (NMDS) ordination plot of bacterial communities based on pairwise Bray–Curtis distances in three Arctic birds (snow buntings, sanderlings, and pink‐footed geese). Circles indicate the species groups which were displayed by ellipses enclosing all points in each group using the “ordiellipse” function in the vegan R package

The NMDS plot shows that the bacterial communities of pink‐footed goose were clustered significantly away from those of sanderling and snow bunting (pink‐footed goose versus sanderling, PERMANOVA, *p* = .003; snow bunting versus sanderling, PERMANOVA, *p* = .002) while bacterial communities were not significantly clustered separately for sanderling and snow bunting (sanderling versus snow bunting, PERMANOVA, *p* = .134) (Figure 5).

**Figure 6:**
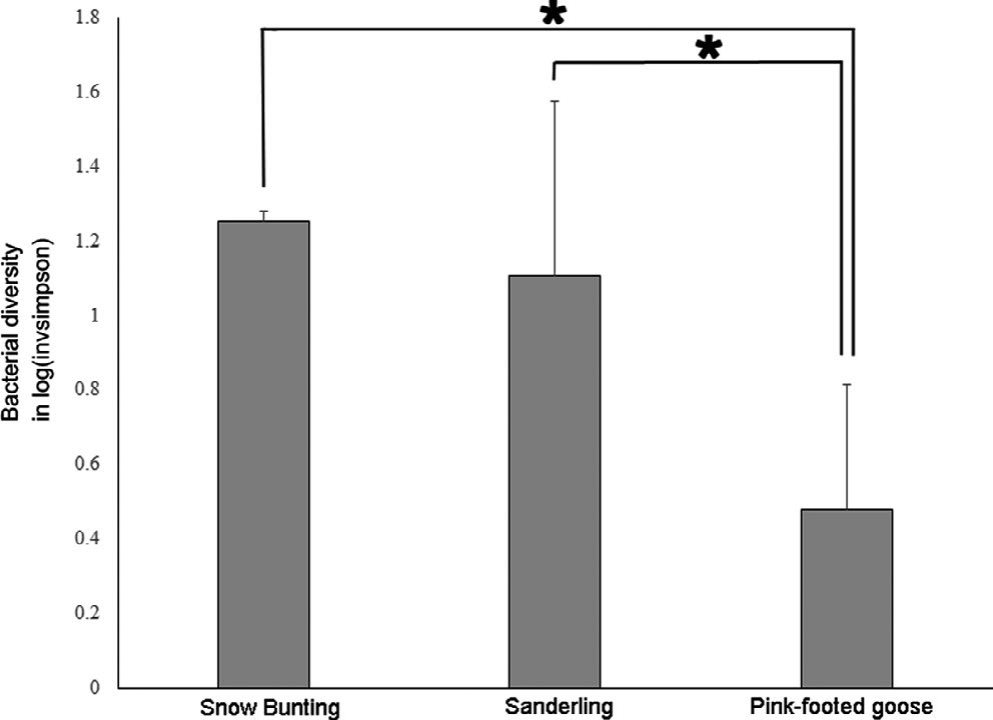
Bacterial diversity (invsimpson index) in the bird fecal microbiota in three Arctic birds (snow buntings, sanderlings, and pink‐footed geese). Asterisks indicate statistical significance (*p* < .05)

The analysis of bacterial diversity revealed significant differences in the invsimpson index between bird species (one‐way ANOVA, *F* = 6.54, *p* = .01). Post hoc tests showed that snow buntings and pink‐footed geese were different (Tukey's test, t = 2.84, *p* = .04) and that sanderlings and pink‐footed geese were different (t = 3.31, *p* = .02), while snow buntings and sanderlings were not significantly different from each other (t = 0.63, *p* = .80) (Figure 6).

**Figure 7:**
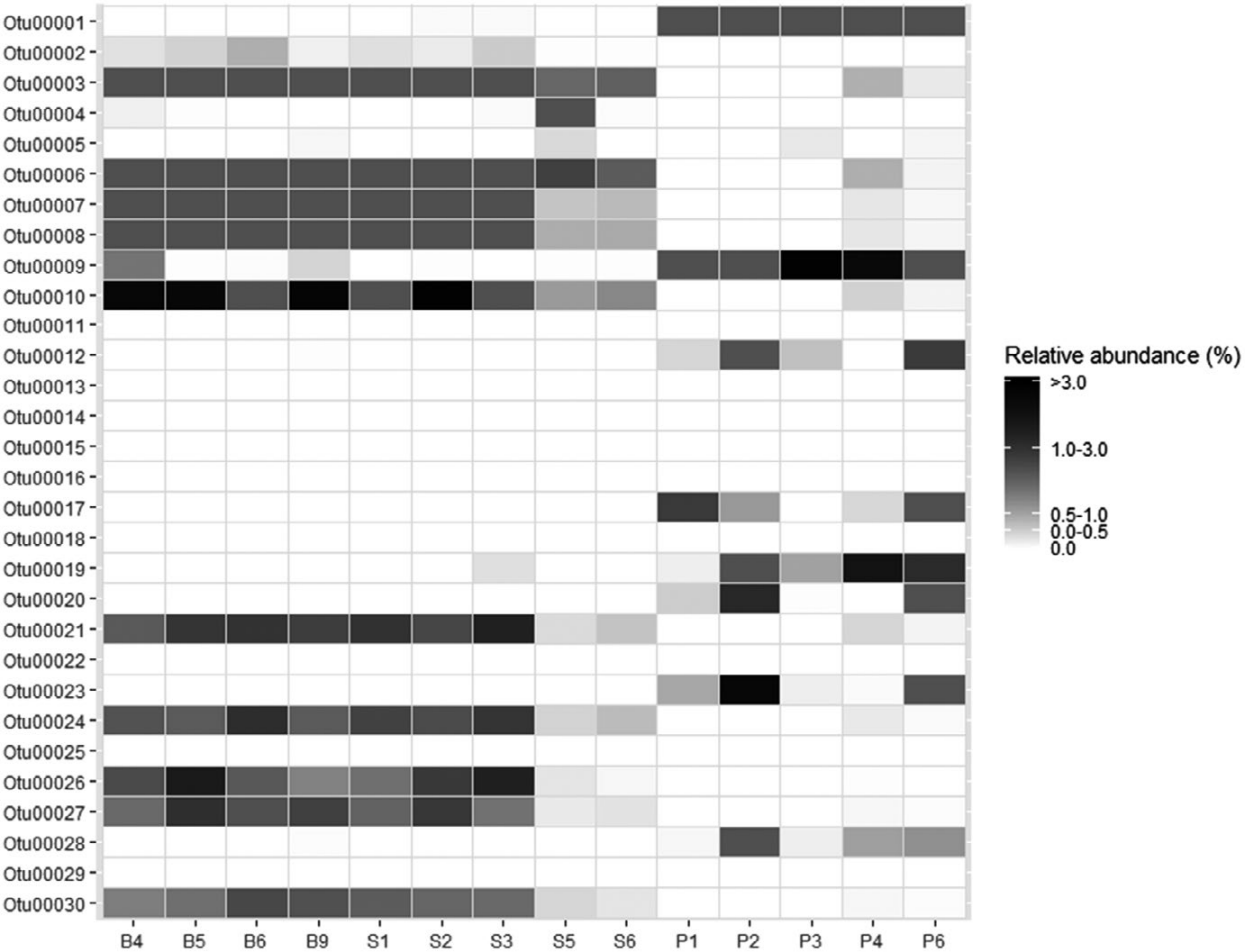
Heat map displaying the relative abundance of dominant OTUs (B4, B5, B6, and B9—snow bunting; S1, S2, S3, S5, and S6—sanderling; P1, P2, P3, P4, and P6—pink‐footed goose)

We further investigated the dominant bacterial OTUs in each sample. The 30 most abundant OTUs from the average of all samples were combined (Figure 7). Overall, the most abundant single OTU (OTU0001: *Pseudomonas unclassified,* phylum: Proteobacteria) was found only among the pink‐footed goose samples. However, four OTUs (OTU0003 (*Streptococcus infantis,* phylum: Firmicutes), OTU0006 (*Preveotella melaninogenica,* phylum: Bacteroidetes), OTU00007 (*Veillonella unclassified,* phylum: Firmicutes), and OTU00008 (*Veillonella unclassified*, phylum: Firmicutes) were abundant in snow bunting and sanderling samples but mostly absent from the pink‐footed goose samples.

PICRUSt was performed to predict the three avian gut microbiome functions based on the 16S rRNA gene sequences. Overall, environmental information processing and membrane transport were the most abundant functions (4.76% in snow buntings; 5.48% in sanderling; 10.71% in pink‐footed geese). Carbohydrate metabolism and amino acid metabolism followed (4.76% in snow buntings; 5.11% in sanderling; and 6.86% in pink‐footed geese) (Appendix S1). The predicted functions and the comparisons among the three arctic bird species were provided in the Appendix S2.

## 4 DISCUSSION

Our results showed that the three Arctic birds have interspecific variations in their fecal microbiota. NMDS plots revealed that snow buntings and sanderlings were similar, while the pink‐footed goose was distant from the other two species. Also, the fecal bacterial structures were characterized with the feeding diet habits of their host species. Snow buntings and sanderlings consume insects during this season and share common diets while pink‐footed geese have a different feeding habit. In our study area, snow buntings and sanderlings were reported to eat insects, while geese had plant food sources near the water (Lee, 2018).

Although the bacterial communities of snow buntings and sanderlings were similar each other, sanderlings had more dispersed values compared to snow buntings in the NMDS plot. It can be explained by the differences in their feeding habits. Snow buntings are altricial birds that depend on their parents for food (Maher, 1964), enabling parental influence on nestling's gut microbiota through prey selection and transfer of saliva. In contrast, sanderlings are precocial birds (Parmelee & Payne, 1973) that chicks leave the nest soon after hatch and often forage independently. Considering that microbial colonization of young bird guts occurs through various routes (Grond et al., 2018), the broader range of food source of sanderling chicks in early breeding stage might be responsible for the dispersed microbial range of sanderlings in NMDS plot. Another possible explanation would be related to their mating systems. According to the previous reports, snow buntings are monogamous (Lyon, Montgomerie, & Hamilton, 1987) while sanderlings had wide ranges of mating strategies even within the population from polyandry to polygyny (Reneerkens. van Veelen, van der Velde, Luttikhuizen, & Piersma, 2014). Microorganisms can be transmitted during copulation in birds and polygamous birds may have higher bacterial diversity (Lee, 2015). Because our samples were collected from male birds in the two species, our results could be affected by their mating behavior. Thus, the complex social system could be a factor to affect the dispersed values in sanderlings compared to the ones in snow buntings.

Our findings also revealed that insectivorous snow buntings and sanderlings had higher fecal bacterial diversity values than the herbivorous pink‐footed geese. In a mammal study, gut bacterial diversity varied according to host diet, from carnivores to omnivores to herbivores (Ley, Hamady, et al., 2008). We think that insectivorous birds would be expected to consume wider variety of food items, including diverse bacterial species, than herbivorous birds. Thus, the higher level of bacterial diversity in snow buntings and sanderlings compared with that in pink‐footed geese may be related to the food types.

In the fecal bacteria of snow buntings, the dominant phyla were Firmicutes (51.62%) and Bacteroidetes (28.19%) accounting for approximately 80% of the phyla. When compared with previous studies in other Passeriformes birds, Proteobacteria, Firmicutes, Bacteroidetes, Actinobacteria, and Tenericutes were reported to be dominant (Table 2). The phylum Firmicutes was the most dominant in this study, which is concordant with house sparrow and finch studies (Mirón et al., 2014; Ochman et al., 2010). Additionally, the phylum Bacteroidetes was the second most dominant in our results, and this taxon was found in barn swallows (Kreisinger, Cizkova, Kropackova, & Albrecht, 2015). In the genus level, Pseudomonas (Phylum: Proteobacteria) was the most abundant in all groups, but it is due to relatively higher percentage in pink‐footed goose (66.17%). In other bird groups, less than 0.1% of Pseudomonas was observed. In contrast, Prevotella (Phylum: Bacteroidetes) and Streptococcus (Phylum: Firmicutes) were the most abundant genera in snow bunting and sanderling (Figure 4).

**Table 2:** Previous and current studies conducted on the fecal microbiota of Passeriformes bird taxa (family and species)

Family	Species	Average percentage of dominant phylum	Feeding type (diet category)	References
Paridae	Great tit *Parus major*	Proteobacteria (42.23%) Tenericutes (19.28%) Firmicutes (16.84%) Actinobacteria (12.72%)	Omnivorous	Kropáčková et al., 2017
Thraupidae	Vampire ground finch *Geospiza septentrionalis*	Firmicutes (50%) Proteobacteria (40%) Actinobacteria (8%)	Blood drinking	Michel et al., 2018
Passeridae	*House sparrow *Passer domesticus*	Firmicutes Proteobacteria	Omnivorous	Mirón et al., 2014
Hirundinidae	*Barn swallow *Hirundo rustica*	Proteobacteria Firmicutes Actinobacteria Bacteroidetes	Insectivorous	Kreisinger et al., 2015
Turdidae	*Swainson's Thrush *Catharus ustulatus*	Proteobacteria Firmicutes Actinobacteria	Insectivorous	Lewis et al., 2017
	*Wood Thrush *Hylocichla mustelina*	Proteobacteria Firmicutes Actinobacteria	Insectivorous	
Mimidae	*Gray catbird *Dumetella carolinensis*	Proteobacteria Firmicutes Actinobacteria	Insectivorous	
Calcariidae	Snow bunting *Plectrophenax nivalis*	Firmicutes(51.62%) Bacteroidetes (28.19%) Actinobacteria (6.53%) Proteobacteria (5.73%)	Insectivorous	This study

* Dominant phyla without a percentage are marked in their abundance order.

In this study on sanderlings, Firmicutes and Bacteroidetes were the most abundant phyla, accounting for over 89% of the total (Figure 3). In previous studies on Charadriiformes, the fecal microbiota of sanderlings mainly harbored Proteobacteria, Fusobacteria, Firmicutes, Bacteroidetes, and Actinobacteria (Risely, Waite, Ujvari, Klaassen, & Hoye, 2017) (Table 3). Although more evidence is needed for the functional roles of microbes, Firmicutes could be related to the insect‐feeding habits of the host birds, and they may contribute to the digestion of the insect food sources of proteins, fats, and carbohydrates. Snow buntings and sanderlings feed mainly on insects under similar breeding environments. Thus, the two species may require similar digestive functions, at least during breeding.

**Table 3:** Previous and current studies conducted on Charadriiformes taxa (family and species)

Family	Species	Average percentage of dominant phylum	Feeding type (diet category)	References
Scolopacidae	Red‐necked stint *Calidris ruficollis*	Proteobacteria (33%) Fusobacteria (17%) Firmicutes (14%)	Omnivorous	Risely et al., 2017
Laridae	Western gull *Larus occidentalis*	Firmicutes (36.5%) Proteobacteria (23.6%) Bacteroidetes (16.1%) Actinobacteria (8%)	Omnivorous	Cockerham et al., 2019
	*European herring gull *Larus argentatus*	Firmicutes Actinobacteria Bacteroidetes Proteobacteria Cyanobacteria	Omnivorous	Fuirst, Veit, Hahn, Dheilly, & Thorne, 2018
Scolopacidae	Sanderling *Calidris alba*	Proteobacteria (76.47%) Firmicutes (12.60%) Bacteroidetes (1.84%) Actinobacteria (1.45%)	Aquatic invertebrates	This study

* Dominant phyla without a percentage are marked in their abundance order.

The dominant phyla of the pink‐footed goose were Firmicutes (49.09%), Bacteroidetes (31.59%), and Proteobacteria (9.33%) in this study. Firmicutes and Proteobacteria were found in other geese, such as white‐fronted goose and bar‐headed goose (Wang et al., 2016;Yang et al., 2016) (Table 4). Bacteroidetes was also commonly found in other studies in Anseriformes. Bacteroidetes are known to assist in the decomposition of polysaccharides, cellulose, and other complex polymers (Thomas, Hehemann, Rebuffet, Czjzek, & Michel, 2011).

**Table 4:** Previous and current studies conducted on Anseriformes taxa (family and species)

Family	Species	Average percentage of dominant phylum	Feeding type (diet category)	References
Anatidae	Bar‐headed goose *Anser indicus*	Firmicutes (58.33%) Proteobacteria (30.67%) Actinobacteria (7.33%) Bacteroidetes (3.33%)	Herbivorous	Wang et al. (2016)
	White‐fronted goose *Anser albifrons*	Firmicutes (49.70%) Proteobacteria (23.80%) Acidobacteria (10.30%) Bacteroidetes (3.80%)	Herbivorous	Yang et al. (2016)
	Bean goose *Anser serrirostris*			
	Swan goose *Anser cygnoides*			
	Northern pintail *Anas acuta*	Firmicutes (33.7%) Proteobacteria (32.7%) Bacteroidetes (13.8%) Fusobacteria (11.6%)	Omnivore	Hird, Ganz, Eisen, and Boyce (2018)
	American wigeon *Mareca americana*			
	Green‐winged teal *Anas carolinensis*			
	Northern shoveler *Spatula clypeata*			
	Mallard *Anas platyrhynchos*			
	Pink‐footed geese *Anser brachyrhynchus*	Firmicutes (49.09%) Bacteroidetes (31.59%) Proteobacteria (9.33%) Actinobacteria (4.95%)	herbivorous	This study

According to a recent study in our study area (Lee, 2018), snow bunting and sanderling were observed to breed in the same study area during the summer in July, pink‐footed geese were recorded to molt there, and breeding was not confirmed in 2016 and 2017 (also by aerial survey in 2008 and 2009 in Boertmann et al.’s study (Boertmann et al., 2015)). It is known that herbivorous b**i**rd guts are often dominated by members of the phylum Bacteroidetes that can assist in the decomposition of polysaccharides, cellulose, and other complex polymers (Thomas et al., 2011) while carnivorous bird species guts are dominated by Proteobacteria and Firmicutes (Blanco, 2014;Grond et al., 2014;Ryu et al., 2014). Because we did not conduct the survey for the whole breeding periods, it is not clear exactly whether geese were breeding or not, but geese appeared to be nonbreeding individuals in the molt stage during our field survey in 2017, as previous studies reported. Molting in birds requires large amounts of energy to produce new feathers and to maintain essential physiological functions. Because of the limited food supply during the molting season, a lack of nutrition will also occur with changes in the gut microbiota (Lee et al., 2019). Therefore, we expect that the breeding status and the stage of molting could affect the distinct bacterial compositions in the pink‐footed goose.

A heat map (Figure 7) illustrating the most abundant OTUs in each bird species showed that the most abundant single OTU (OTU00001—Pseudomonas unclassified) was found only among the pink‐footed goose samples. The fecal microbiota of the pink‐footed goose is dominated by pseudomonas, whose membranes are known to have the ability to hydrolyze phytate and degrade starch in soils, they are known to improve plant phosphorus availability (Maougal et al., 2014)**.** The pink‐footed goose is the most common species of goose and herbivores that utilize both the green and root parts of plants (Fox et al., 2006). However, four OTUs (OTU00003 (*Streptococcus infantis*), OTU00006 (*Prevotella melaninogenica*), OTU00007 (*Veillonella unclassified*), and OTU00008 (*Veillonella unclassified*)) were abundant in snow bunting and sanderling samples but mostly absent from the pink‐footed goose samples. Most of the bacteria detected in snow bunting and sanderling are lactic acid bacteria (Streptococcus). Lactic acid bacteria dominate the fecal microbiota of insectivores. One of their main functions in the human digestive tract is carbohydrate metabolism (Hammes & Hertel, 2006), and a similar function is expected in birds.

Additionally, we employed the PICRUSt analysis to infer potential gene profiles from 16S rRNA sequencing. This analysis showed the predicted functional pathways in the three species. Metabolic pathways (environmental information processing and membrane transport, carbohydrate metabolism, and amino acid metabolism) were commonly abundant, possibly correlating with the demand for breeding and molting. However, the results should be carefully understood due to the limitation of the predictions using reference data. In summary, through the application of a high‐throughput DNA sequencing approach, this study identified variation between the microbiota of three migratory birds. Similarity was observed in the fecal microbiota of two ecologically different species breeding in the same habitat during the summer season in the Arctic. Firmicutes and Bacteroidetes dominated the fecal microbiota of snow bunting and sanderling, while Proteobacteria and Firmicutes dominated in the pink‐footed goose. Although host phylogeny and digestive physiology may cause these differences, diet could potentially play a major role in determining the final microbial composition of individual seabird species.

One challenge of studying wild birds under natural conditions is disentangling the large number of factors that can influence host microbial communities. In this study, there was a limitation that the breeding status of those birds was not well investigated. Nevertheless, our study will shed more light on the interaction between animal behavior and the fecal microbiota. Our study also provides basic information that might be used in future studies to better understand the avian gut microbiota and might be expanded to investigate how the gut microbiota affects body conditions, the immune system, and the behavior of migratory birds in Arctic. How the bacteria coordinate in the gut microbiota and how these bacteria interact with their hosts need to be clarified. Thus, more topics in the ecology and physiology of the gut microbiota in birds are very attractive fields for study.

We further suggest collecting fecal samples among the different groups of birds through different breeding stages that have potential influences of the host species and diet on the microbial community assemblages. As the gut microbiota may coevolve with diet selection, analyzing these microbes may help us understand migratory birds’ preference for natural food in the Arctic and provide new perspectives for bird conservation. Nevertheless, little is known about the gut microbiota or its functions in arctic migratory birds. This study may be an early attempt to examine the gut microbiota of breeding wild birds under natural dietary conditions in the high Arctic region that provides the basis for future comparative studies with the same species that are confined to other habitats in other parts during wintering periods.

In the future studies, it is necessary to examine the effects of diet on the host gut microbiota in the closely related bird species, excluding the phylogenetic effects. It will be interesting to test the hypothesis in the same genus species with different feeding behaviors.

## COMPETING INTERESTS STATEMENT

The authors have declared that no competing interests exist.

## AUTHOR CONTRIBUTIONS

**Hyunjun Cho:** Formal analysis (equal); Methodology (equal); Writing‐original draft (equal); Writing‐review & editing (equal). **Won Young Lee:** Conceptualization (lead); Data curation (lead); Formal analysis (equal); Funding acquisition (equal); Investigation (lead); Methodology (lead); Project administration (lead); Resources (lead); Supervision (lead); Writing‐original draft (equal); Writing‐review & editing (equal).

## Supporting information

Appendix S1

Appendix S2

## Data Availability

The data that support the findings of this study are openly available in MG‐RAST server under project “arctic bird faecal microbiota” (https://www.mg‐rast.org/linkin.cgi?project=mgp90221).
