# Supplementary material for: Interspecific comparison of the fecal microbiota structure in three Arctic migratory bird species
Source: Ecol Evol. 2020 May 18;10(12):5582–94. doi: 10.1002/ece3.6299 (PMC7319242; doi:10.1002/ece3.6299)
Supplement: Supplementary file 1 — Appendix S1 [file ECE3-10-5582-s001.docx]

**Supplementary Information Appendix S1. The predicted function by PICRUSt to estimate the microbial functions in the gut microbiota metagenomics profiles in the KEGG pathways at level 2**

**
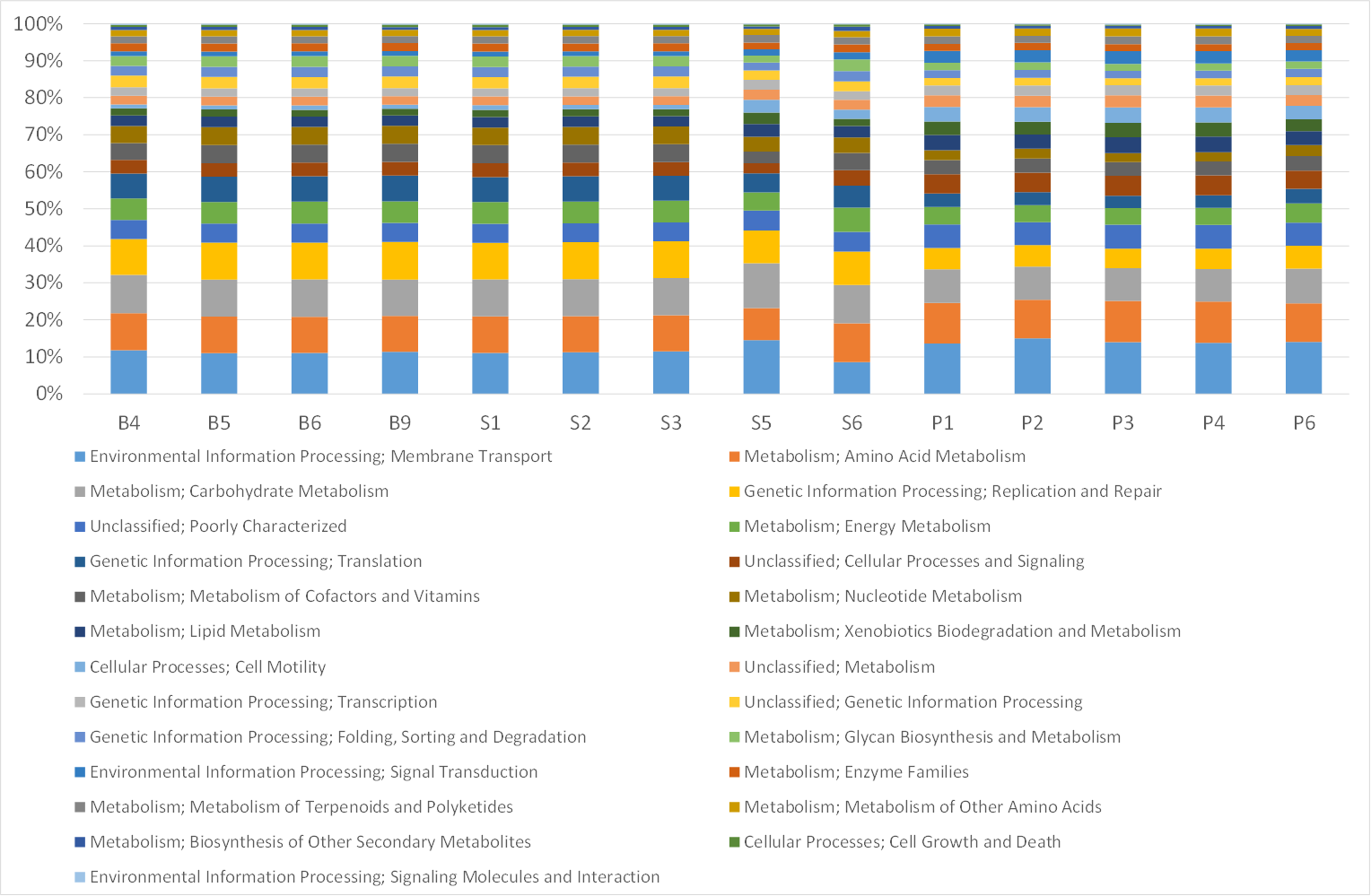
**

**Figure 1. The predicted functions in all individuals that you used in the microbial analysis (B4-9: snow bunting, S1-6: sanderling, P1-6: pink-footed goose) in the KEGG pathways at level 2**

**
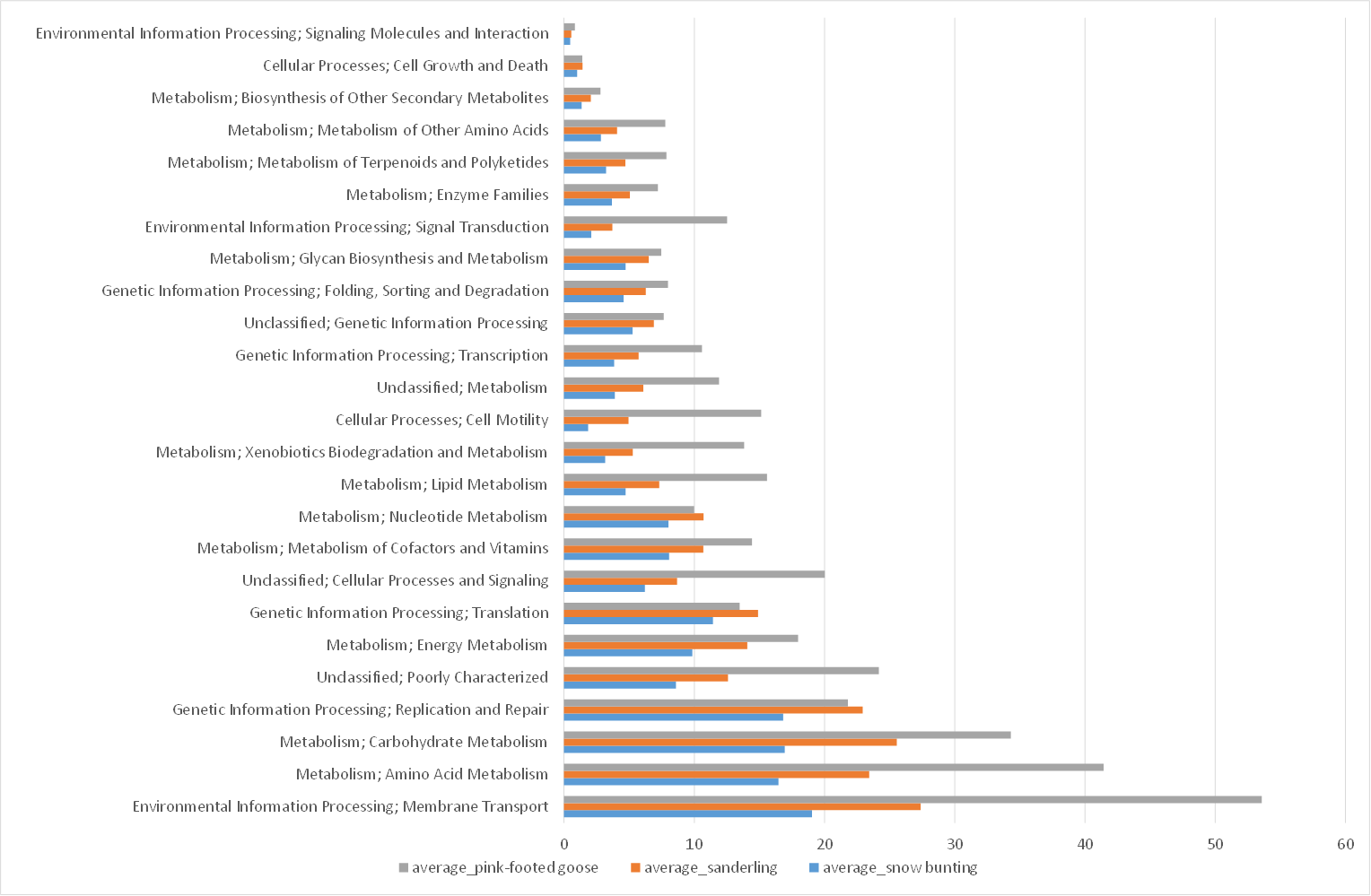
**

**Figure 2. The predicted functions of the average values in the three species (snow bunting, sanderling and pink-footed goose) in the KEGG pathways at level 2**
