# Supplementary material for: Interspecific comparison of the fecal microbiota structure in three Arctic migratory bird species
Source: Ecol Evol. 2020 May 18;10(12):5582–94. doi: 10.1002/ece3.6299 (PMC7319242; doi:10.1002/ece3.6299)
Supplement: Supplementary file 2 — Appendix S2 [file ECE3-10-5582-s002.docx]

**Supplementary Information Appendix S2. Comparisons among the three arctic bird species (snow bunting, sanderling and pink-footed goose) in the gut microbiota metagenomics profiles in the KEGG pathways at level 2 (significances were tested by (p<0.05, Welch’s two sample t-test)**

**Table 1. Comparison between snow bunting and pink-footed goose**

| KEGG level | KEGG pathway term | p-value |
| --- | --- | --- |
| 2 | Glycan Biosynthesis and metabolism | <0.01 |
| 2 | Xenobiotics biodegradation and metabolism | <0.01 |
| 2 | Lipid metabolism | <0.01 |
| 2 | Enzyme families | <0.01 |
| 2 | Carbohydrate metabolism | <0.01 |
| 2 | Amino acid metabolism | <0.01 |
| 2 | Metabolism of other amino acids | <0.01 |
| 2 | Nucleotide metabolism | <0.01 |
| 2 | Metabolism of cofactors and vitamins | <0.01 |
| 2 | Energy metabolism | 0.013 |
| 2 | Circulatory system | <0.01 |
| 2 | Nervous system | <0.01 |
| 2 | Digestive system | <0.01 |
| 2 | Immune system | <0.01 |
| 2 | Environmental adaptation | 0.012 |
| 2 | Cell mortility | <0.01 |
| 2 | Cell growth and death | 0.044 |
| 2 | Folding, sorting and degradation | <0.01 |
| 2 | Replication and reproduction | <0.01 |
| 2 | Translation | <0.01 |
| 2 | transcription | 0.038 |

**Table 2 Comparison between sanderling and pink-footed goose**

| KEGG level | KEGG pathway term | p-value |
| --- | --- | --- |
| 2 | Nucleotide metabolism | <0.01 |
| 2 | Metabolism of other amino acids | <0.01 |
| 2 | Lipid metabolism | <0.01 |
| 2 | Xenobiotics biodegradation and metabolism | <0.01 |
| 2 | Carbohydrate metabolism | <0.01 |
| 2 | Energy metabolism | <0.01 |
| 2 | Carbohydrate metabolism | <0.01 |
| 2 | Glycan biosynthesis and metabolism | <0.01 |
| 2 | Enzyme families | 0.011 |
| 2 | Amino acid metabolism | 0.022 |
| 2 | Nervous system | <0.01 |
| 2 | Digestive system | <0.01 |
| 2 | Circulatory system | <0.01 |
| 2 | Cell growth and death | <0.01 |
| 2 | Cell mortility | <0.01 |
| 2 | Replication and reproduction | <0.01 |
| 2 | Translation | <0.01 |
| 2 | Folding, sorting and degradation | <0.01 |
| 2 | Transcription | <0.01 |

**Table 3. Comparison between snow bunting and sanderling**

| KEGG level | KEGG pathway term | p-value |
| --- | --- | --- |
| 2 | Environmental adaptation | 0.029 |
